# β‐Amyrin synthase from *Conyza blinii* expressed in *Saccharomyces cerevisiae*

**DOI:** 10.1002/2211-5463.12299

**Published:** 2017-09-06

**Authors:** Rong Sun, Shan Liu, Zi‐Zhong Tang, Tian‐Run Zheng, Tao Wang, Hui Chen, Cheng‐Lei Li, Qi Wu

**Affiliations:** ^1^ College of Life Science Sichuan Agricultural University Ya'an China; ^2^ College of Biological and Chemical Engineering Panzhihua University China

**Keywords:** *Conyza blinii* H.Lév., conyzasaponins, *Saccharomyces cerevisiae*, β‐amyrin synthase

## Abstract

*Conyza blinii* H.Lév. is a widely used medicinal herb in southwestern China. The main pharmacological components of *C. blinii* are a class of oleanane‐type pentacyclic triterpene glycosides known as conyzasaponins, which are thought to be synthesized from β‐amyrin. However, no genes involved in the conyzasaponin pathway have previously been identified. Here, we identify an oxidosqualene cyclase (OSC), a β‐amyrin synthase, which mediates cyclization of 2,3‐oxidosqualene to yield β‐amyrin. Ten OSC sequences were isolated from *C. blinii* transcript tags. Phylogenetic analysis was used to select the tag Cb18076 as the putative β‐amyrin synthase, named *CbβAS*. The open reading frame of *CbβAS* is 2286 bp and encodes 761 amino acids. Its mature protein contains the highly conserved motifs (QXXXGXW/DCTAE) of OSCs and (MWCYCR) of β‐amyrin synthases. Transcription of *CbβAS* was upregulated 4–24 h after treatment of the seedlings of the *C. blinii* cultivar with methyl jasmonate. Furthermore, expression of *CbβAS* in *Saccharomyces cerevisiae* successfully yielded β‐amyrin. The chemical structures and concentrations of β‐amyrin were confirmed by GC‐MS/MS. The target yeast ultimately produced 4.432 mg·L^−1^ β‐amyrin. Thus, CbβAS is an OSC involved in conyzasaponin biosynthesis.

**Abbreviations**
*βAS*: β‐Amyrin synthase
CAS: cycloartenol synthase
DS: dammarenediol synthase
GAPDH: glyceraldehyde‐3‐phosphate dehydrogenase
GO: Gene Ontology
HMG‐CoA: 3‐hydroxyl‐3‐methylglutaryl‐CoA
IPP: isopentenyl pyrophosphate
LUS: lupeol synthase
MeJA: methyl jasmonate
MRM: multireaction monitor
OSC: oxidosqualene cyclase
Pfam: protein family
*PtBS*: *Polygala tenuifolia* Willd. *βAS*
SC‐U: SC minimal media lacking uracil
Unigenes: universal gene

*Conyza blinii* H.Lév. is a medicinal herb distributed in southwestern China (Sichuan, Yunnan, and Guizhou provinces). It is well known for its treatment of bronchitis cough and inflammatory diseases. The entirety of the plant can be medicinally prepared and the highest accumulate of its secondary metabolites are conyzasaponins (3.0% w/w, of dry weight). Seventeen conyzasaponins have been isolated from the ethanol extract of *C. blinii*, of which all are oleanane‐type saponins 1, 2, 3.

The current studies suggest that the synthesis of saponins is divided into four stages: first, the biosynthesis of isopentenyl pyrophosphate (IPP) and dimethylallyl pyrophosphate; second, the biosynthesis of 2, 3‐oxidosqualene; third, the biosynthesis of the basic backbone; fourth, the modification of the backbone ring. The third step is a branch. This step is catalyzed by oxidosqualene cyclases (OSCs) and resulted in multiple saponins backbones, including oleanane type, lupeol type, ursane type. Many OSCs have been reported to have multifunctional activities that can biosynthesize more than one saponins backbone 4, 5, 6. However, one of the OSCs, β‐amyrin synthase, controls flux toward the oleanane‐type backbone (β‐amyrin).

β‐Amyrin synthase (*βAS*) has been isolated and characterized from many high plants with abundant oleanane‐type saponins. Jin *et al*. 7 isolated a *Polygala tenuifolia* Willd. *βAS* (*PtBS*) that contained a 2289‐bp reading frame. Expression of *PtBS* in the yeast led to the production of β‐amyrin as the sole product. The *βAS* from *Artemisia annua* expressed in *Saccharomyces cerevisiae* with manipulation of 3‐hydroxyl‐3‐methylglutaryl‐CoA (HMG‐CoA) reductase and lanosterol synthase produced levels of 6 mg·L^−1^ culture of β‐amyrin 8. Huang *et al*. 9 transformed *Panax japonicus βAS* into rice to produce ‘ginseng rice’, which was capable of producing oleanane‐type sapogenin.

*Saccharomyces cerevisiae* was widely used as an excellent host for the production of medicinal terpenes because of its mevalonate pathway and safety. Paddon *et al*. 10 have semisynthesized artemisinin in *S. cerevisiae*. The production of artemisinic acid, a precursor of artemisinin, reached a level of 25 g·L^−1^. This technology may increase antimalarial treatments in the developing world. Engels *et al*. 11 produced 8.7 ± 0.85 mg·L^−1^ taxadiene by using coexpression of codon‐optimized taxadiene synthase, truncated HMG‐CoA reductase, the UPC2‐1 transcription factor gene, and geranylgeranyl diphosphate synthase in *S. cerevisiae*. Furthermore, Han *et al*. 12 combined biosynthesis of protopanaxadiol in *S. cerevisiae* via coexpression of dammarenediol synthase (DS) and cytochrome P450 monooxygenase. After 2‐day induction, the engineering yeast yielded 17.32 μg·g^−1^ (FW) protopanaxadiol. In this study, we express a β‐amyrin synthase gene of *C. blinii* in *S. cerevisiae* to produce β‐amyrin. The putative biosynthesis pathway for β‐amyrin in native yeast is shown in Fig. 1.

**Figure 1:**
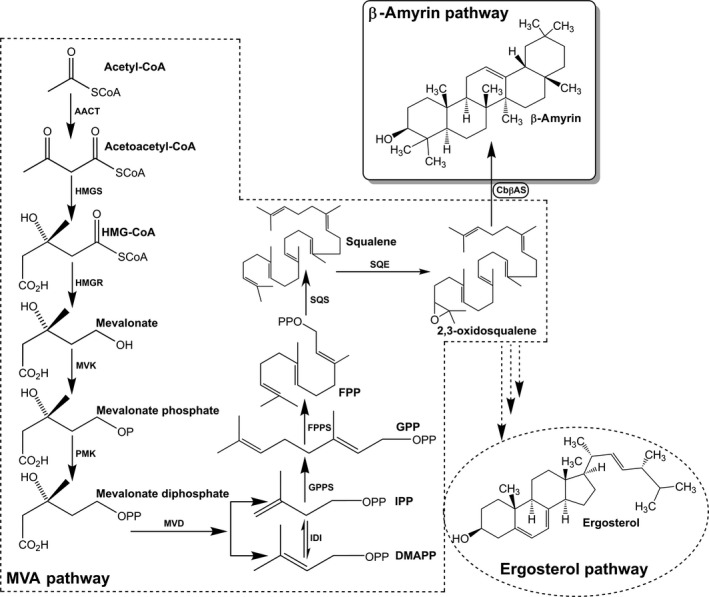
β‐Amyrin biosynthesis pathway engineered in yeast. The *CbβAS* cyclizes 2,3‐oxidosqualene to β‐amyrin. The enzymes involved in this pathway: AACT, acetyl coenzyme A acetyltransferase; HMGS, 3‐hydroxy‐3‐methylglutaryl coenzyme A synthase; HMGR, 3‐hydroxy‐3‐methylglutaryl coenzyme A reductase; MVK, mevalonate kinase; PMK, phosphor mevalonate kinase; MVD, mevalonate diphosphate decarboxylase; IDI, IPP isomerase; GPPS, geranyl diphosphate synthase; FPPS, farnesyl diphosphate synthase; SQS, squalene synthase; SQE, squalene epoxidase; CbβAS, *Conyza blinii* β‐amyrin synthase.

Here, we cloned and characterized CbβAS, a β‐amyrin synthase that catalyzes the cyclization of oxidosqualene in the biosynthesis of conyzasaponins. Ectopic expression of *CbβAS* in INVSc1 yeast successfully yielded β‐amyrin. The results confirm that *CbβAS* is a β‐amyrin synthase.

## Materials and methods

### Plant material

*Conyza blinii* used for gene cloning were collected in 2014 from Panzhihua, Sichuan, China. *C. blinii* multiple shoots (differentiated by our laboratory) were induced in 1/2 MS culture medium, which containing 0.1 mg·L^−1^ 1‐naphthylacetic acid to obtain aseptic seedling. Seedlings were grown with light and constant temperature at 24 °C. Two months later, plants were treated with either the 100 μmol·L^−1^ methyl jasmonate (MeJA) or the control ethanol by spraying. Leaves were collected at 0, 2, 4, 8, 12, and 24 h after treatment and then stored at −80 °C.

### Cloning of *CbβAS*

Ten OSC genes were discovered from the *C. blinii* transcriptome annotation library 13. The phylogenetic analysis was used to select the *βAS* gene. OSC protein sequences including βAS, DS, CAS, and LUS were retrieved from NCBI. The sequence alignments were performed using clustalw program (http://clustalw.ddbj.nig.ac.jp). The mega 5.05 software 14 was used to build the phylogenetic tree with neighbor‐joining method and 1000 bootstrap replications.

According to the selected sequence, specific primers BAS1 and BAS2 (Table 1) were designed. The 50 μL reaction system included 25 μL PrimeSTAR Max DNA Polymerase Premix (2×) (TaKaRa, Kyoto, Japan), 10 pmol BAS1, 10 pmol BAS2, 100 ng cDNA, and ddH_2_O. According to the introduction of Max DNA Polymerase, the three‐step PCR program was used to amplify the *CbβAS* gene. PCR products were then purified (TaKaRa MiniBEST Agarose Gel DNA Extraction Kit Ver.4.0) and sequenced (Invitrogen Trading, Shanghai, China). Afterward, the nucleotide sequence and the deduced amino acid sequence were characterized by bioinformatics tools.

**Table 1:** Primers used in this study

Primers	Sequence (5′→3′)
Gene cloning primers	
BAS1	ATGTGGAGAATGAATATAG
BAS2	CTAGATGCGTTTGAGCTTTGG
Quantitative RT‐PCR primers	
GAPDHqF	CGGGATGGCTTTCCGTGTA
GAPDHqR	TTGCCTTCTGATTCCTCCTTGA
BASqF	TTGGCAGTCAAGAGTGGGATG
BASqR	GGAAGGATTGTCTTTGACCTGTGA
*Saccharomyces cerevisiae* expression primers	
BAS3	AAATATgcggccgcATGTGGAGAATGAATATAG
BAS4	TGCtctagaCTAGATGCGTTTGAGCTTTGG

### Quantitative RT‐PCR analysis

Methyl jasmonate‐treated leaves were used as samples for qRT‐PCR analysis. The same amount of RNA from samples was used for reverse transcription into the single‐stranded cDNA according to the PrimeScript RT Reagent Kit with gDNA Eraser (TaKaRa). The housekeeping gene previously published, glyceraldehyde‐3‐phosphate dehydrogenase (GAPDH; GenBank ID: KF027475) 15, was used as the internal control. The qRT‐PCR primers are in Table 1. A 25 μL reaction system with SYBR Premix Ex Taq II (TaKaRa) was used for quantification on a CFX96 Real‐Time PCR Instrument (Bio‐Rad, Hercules, CA, USA). The $2^{-\Delta \Delta C_{T}}$ method 16 was used to calculate differences among gene expression. The experiments were replicated four times.

### Expression of *CbβAS* in *Saccharomyces cerevisiae* INVSc1

The expression vector pYES2/NT B (provided by Zongyun Feng, Sichuan Agricultural University) and the *S. cerevisiae* strain INVSc1 (provided by Zongyun Feng, Sichuan Agricultural University) were used to examine CbβAS function. The open reading frame of *CbβAS* was amplified with primers BAS3 and BAS4 (Table 1). The PCR products were inserted into the *Not*I and *Xba*I restriction sites of the pYES2/NT B vector to construct pYES‐CbβAS recombinant plasmid. The pYES‐CbβAS plasmid was transformed into INVSc1 by electroporation (1.5 kV, 3 ms, 2.5 μF, 200 Ω) 17. After 3 days of growth, single clones of INVSc1 containing pYES‐CbβAS or pYES2/NT B were inoculated in 15 mL of SC minimal media lacking uracil (SC‐U) medium containing 2% glucose. Precultures were grown overnight at 30 °C with shaking at 200 r.p.m. To induce gene expression, the precultures were washed and inoculated into 50 mL of induction medium (SC‐U medium containing 2% galactose) with a starting optical density of 0.4. The cultures were further incubated for 60 h to induce *CbβAS* expression.

### Metabolite extraction for GC‐MS/MS analysis

Extraction of metabolites followed the method previously described by Kirby *et al*. 8 with some modifications. 50 mL of induction cells was centrifuged at 2739 ***g*** for 5 min to obtain a cell pellet. The cells were resuspended in 10 mL 20% KOH/50% EtOH (W/V), and the supernatant was discarded. The mixture was boiled for 10 min. After cooling, metabolites were extracted twice using hexane (15 mL). The extracts were combined and analyzed by GC‐MS/MS.

The GC‐MS/MS analysis was performed by 7890B GC model and 7000C MS model (Agilent, Santa Clara, CA, USA). A 1 μL aliquot of the sample was injected (splitless mode) into a HP‐5MS ultra‐inert column (30 m × 0.25 mm × 0.25 μm) (Agilent). The flow rate of helium was 1.5 mL·min^−1^. The column temperature program was performed using the same method described by Seki *et al*. 18. For the quantification of β‐amyrin, the secondary MS was used. The ion *m/z* 189 and *m/z* 203 were designated as quantitative ion and qualitative ion, respectively. The standard β‐amyrin was purchased from Sigma‐Aldrich (St. Louis, MO, USA).

## Results

### Phylogenetic analysis of OSCs and cloning of *CbβAS*

**Figure 2:**
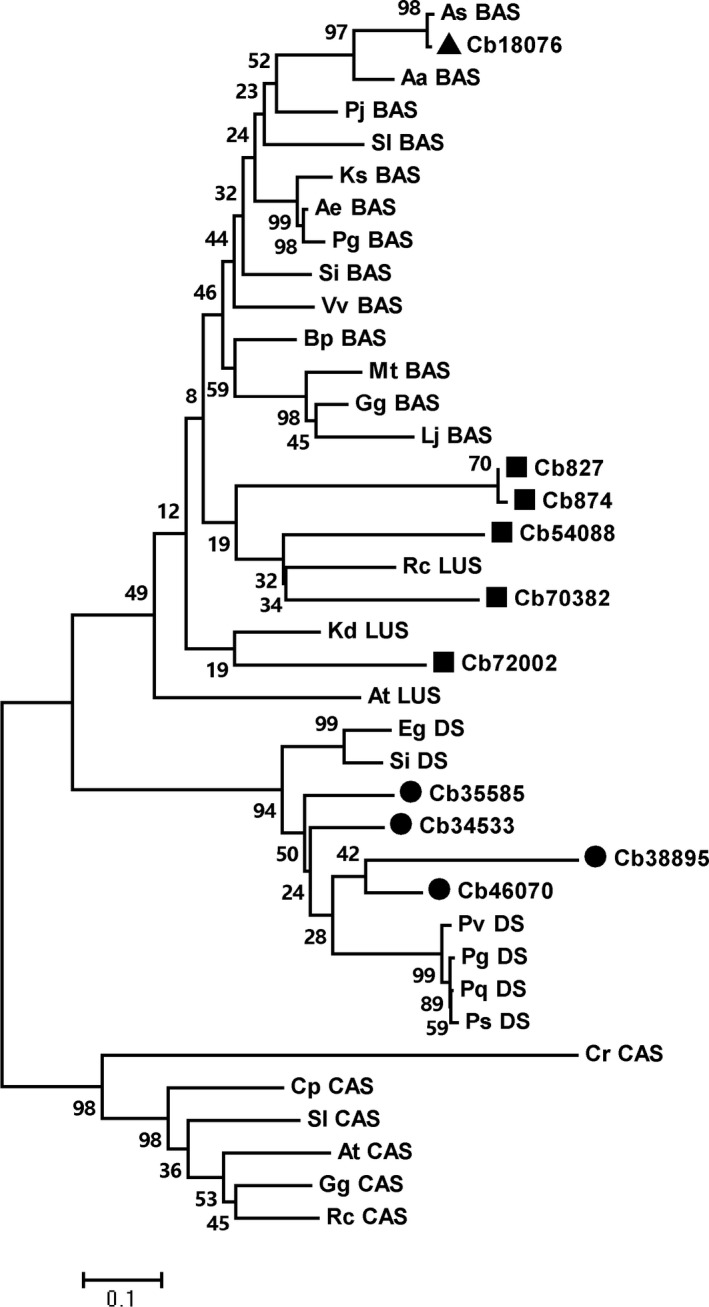
A phylogenetic tree between *Conyza blinii* OSCs and other plant OSCs. The OSCs from *C. blinii* have been marked with triangle, blocks, and circles. The species abbreviations are As, *Aster sedifolius*; Aa, *Artemisia annua*; Pj, *Panax japonicus*; Sl, *Solanum lycopersicum*; Ks, *Kalopanax septemlobus*; Ae, *Aralia elata*; Pg, *Panax ginseng*; Si, *Sesamum indicum*; Vv, *Vitis vinifera*; Bp, *Betula platyphylla*; Mt, *Medicago truncatula*; Gg, *Glycyrrhiza glabra*; Lj, *Lotus japonicus*; Rc, *Ricinus communis*; At, *Arabidopsis thaliana*; Kd, *Kalanchoe daigremontiana*; Eg, *Erythranthe guttata*; Pv, *Panax vietnamensis*; Pq, *Panax quinquefolius*; Ps, *Panax sokpayensis*; Cr, *Chlamydomonas reinhardtii*; Cp, *Cucurbita pepo*.

According to the transcriptome analysis, ten tags corresponded to OSC genes (Table 2). Annotation results showed that six tags were predicted to be β‐amyrin synthase. To further determine the βAS gene, we performed the phylogenetic analysis between these tags and OSCs from other plants. The results revealed that tag Cb18076 was homologous to β‐amyrin synthase from *Aster sedifolius*, which has been reported to only produce β‐amyrin in yeast 19 (Fig. 2). The tags Cb54088, Cb70382, Cb827, and Cb874 were phylogenetically related to *Ricinus communis* LUS 20. Cb72002 was similar to LUS from *Kalanchoe daigremontiana*, which produces lupeol and β‐amyrin in a ratio of 13 : 1 21. In addition, another four tags Cb34533, Cb35585, Cb38895, and Cb46070 were homologous to DS from the *Panax* species, which is involved in the ginsenoside biosynthetic pathway 22, 23. Therefore, we selected the Cb18076 tag as a β‐amyrin synthase gene.

**Table 2:** The tags corresponding to OSC genes and the annotations of them. GO, Gene Ontology; Pfam, protein family

Gene ID	GO annotation	Pfam annotation	SwissProt annotation	Nr annotation
Cb18076	GO:0019745	Prenyltransferase and squalene oxidase repeat	Beta‐amyrin synthase GN = OSCBPY OS = Betula platyphylla (Asian white birch) PE = 1 SV = 1	Beta‐amyrin synthase (*Aster sedifolius*)
	GO:0042300			
Cb34533	GO:0008152	–	Dammarenediol II synthase GN = PNA OS = Panax ginseng (Korean ginseng) PE = 1 SV = 1	OSC2 (*Artemisia annua*)
	GO:0016021			
	GO:0016829			
	GO:0016866			
Cb35585	GO:0008152	Prenyltransferase and squalene oxidase repeat	Dammarenediol II synthase GN = PNA OS = Panax ginseng (Korean ginseng) PE = 1 SV = 1	OSC2 (*A. annua*)
	GO:0016866			
Cb38895	GO:0003824	Prenyltransferase and squalene oxidase repeat	Dammarenediol II synthase GN = PNA OS = Panax ginseng (Korean ginseng) PE = 1 SV = 1	OSC2 (*A. annua*)
Cb46070	GO:0008152	–	Dammarenediol II synthase GN = PNA OS = Panax ginseng (Korean ginseng) PE = 1 SV = 1	OSC2 (*A. annua*)
	GO:0016021			
	GO:0016829			
	GO:0016866			
Cb54088	GO:0008152	–	Beta‐amyrin synthase GN = OSCBPY OS = Betula platyphylla (Asian white birch) PE = 1 SV = 1	PREDICTED: beta‐amyrin synthase‐like (*Fragaria vesca* subsp. *vesca*)
	GO:0016866			
Cb70382	GO:0016104 GO:0042299	–	Lupeol synthase GN = LUS OS = Bruguiera gymnorhiza (Burma mangrove) PE = 1 SV = 1	PREDICTED: beta‐amyrin synthase‐like (*Prunus mume*)
Cb72002	GO:0008152	–	Beta‐amyrin synthase 1 GN = OSCPNY1 OS = Panax ginseng (Korean ginseng) PE = 1 SV = 1	PREDICTED: beta‐amyrin synthase‐like (*F*. *vesca* subsp. *vesca*)
	GO:0016866			
Cb827	GO:0008152	Prenyltransferase and squalene oxidase repeat	Beta‐amyrin synthase GN = OSCBPY OS = Betula platyphylla (Asian white birch) PE = 1 SV = 1	PREDICTED: beta‐amyrin synthase‐like (*F. vesca* subsp. *vesca*)
	GO:0016866			
Cb874	GO:0008152	–	Beta‐amyrin synthase GN = OSCBPY OS = Betula platyphylla (Asian white birch) PE = 1 SV = 1	PREDICTED: beta‐amyrin synthase‐like (*F. vesca* subsp. *vesca*)
	GO:0016866			

**Figure 3:**
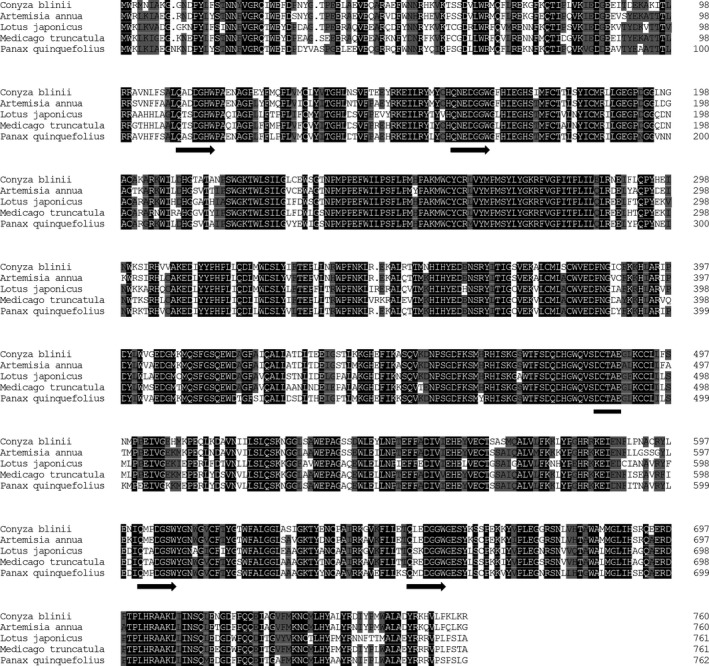
Alignment of the deduced amino acid sequences of *Conyza blinii* βAS and βAS from *Artemisia annua* (ACB87531.1), *Lotus japonicus* (AAO33580.1), *Medicago truncatula* (CAD23247.1), and *Panax ginseng* (BAA33461.1). The 100% homology levels of the residues are shaded in black, and ≥ 75% homology levels of the residues are shaded in gray. The QXXXGXW motifs and DCTAE motif are indicated by arrows and hyphen, respectively.

The cDNA of Cb18076 was cloned and we renamed it as *CbβAS*. The open reading frame of *CbβAS* (GenBank ID: KX907781) was 2286 bp and encoded an 87.7‐kDa protein. The sequence alignment between CbβAS and other plant βAS revealed 85.68% similarity (Fig. 3). The mature protein contained highly conserved motifs (QXXXGXW/DCTAE) of OSCs 24, 25. Its secondary structure was predicted by the SOPMA method (https://npsa-prabi.ibcp.fr/cgi-bin/npsa_automat.pl?page=/NPSA/npsa_sopma.html). The most abundant structures were alpha helices (42.84%), then 31.41% random coils, 15.9% extended strands, and 9.86% beta turns.

### Expression of *CbβAS* gene following treatment by MeJA

**Figure 4:**
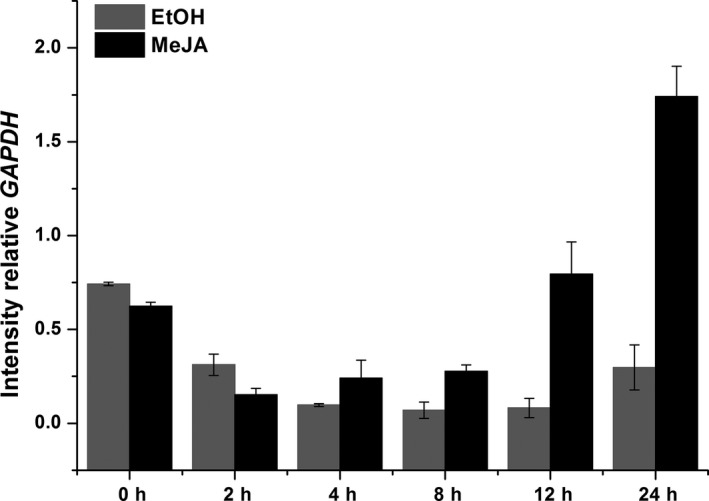
Expression analysis of the *CbβAS* gene in *Conyza blinii* seedling under EtOH and MeJA treatments. The quantitative real‐time PCR assay was used to examine the *CbβAS* relative transcription levels at 0, 2, 4, 8, 12, and 24 h. The expression level of *CbβAS* in no treated seedling was set as control. Standard deviation was calculated by spss software (IBM Corporation, Armonk, NY, USA).

Methyl jasmonate is used as an exogenous elicitor that can enhance the content of secondary metabolites such as saponins 26, 27 and the transcription levels of genes involved in saponins biosynthesis 12, 28. Therefore, to identify whether *CbβAS* gene involved in conyzasaponins pathway, we investigated expression of *CbβAS* after elicitation by MeJA using qRT‐PCR (Fig. 4). The transcript level of *CbβAS* at 24 h was 2.8‐fold higher than at 0 h. Furthermore, MeJA‐treated *CbβAS* transcript levels were six times higher than those of EtOH‐treated *CbβAS* at 24 h. *CbβAS* expression was significantly upregulated by MeJA. The results preliminarily confirm that *CbβAS* is involved in conyzasaponins biosynthetic pathway.

### Functional characterization of *CbβAS*

**Figure 5:**
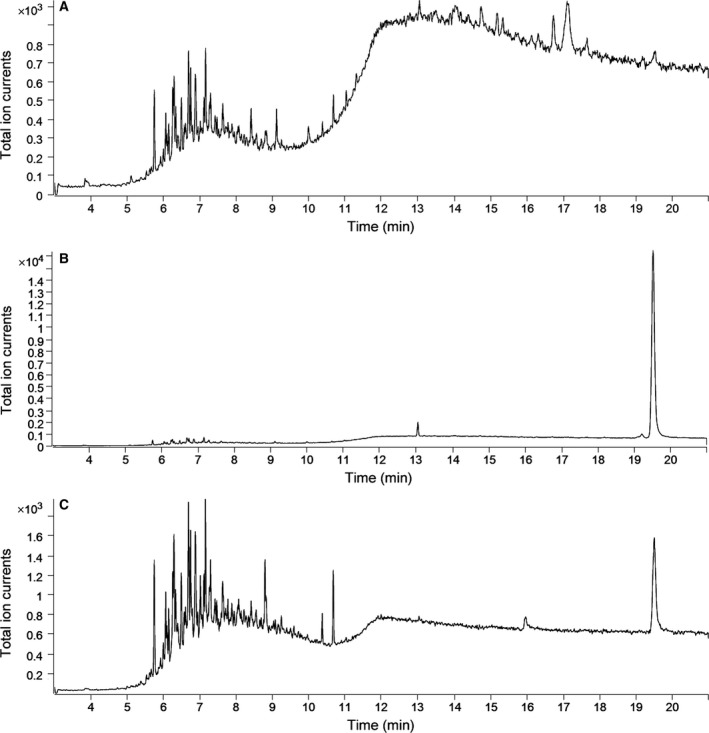
GC chromatograms of yeast extracts. (A) Chromatograms of yeast extracts with an empty pYES2/NT B vector. (B) Chromatograms of standard β‐amyrin. (C) Chromatograms of yeast extracts with pYES‐CbβAS.

**Figure 6:**
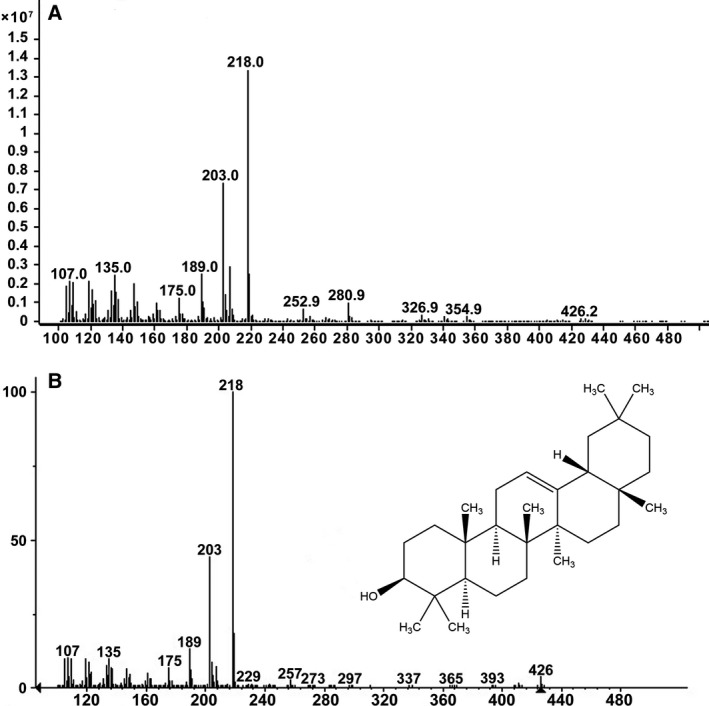
MS spectrum and structure of β‐amyrin. (A) MS spectrum of β‐amyrin produced in pYES‐CbβAS yeast. (B) MS spectrum and structure of the β‐amyrin standard.

To detect the activity of *CbβAS*, the recombinant plasmid pYES‐CbβAS was constructed. The pYES‐CbβAS plasmid was then expressed in INVSc1 under the control of GAL1 promoter. To verify the function of *CbβAS*, the yeast extracts were examined by GC‐MS. The GC retention time showed that at 19.5 min, pYES‐CbβAS strain and standard β‐amyrin appeared a peak, while the pYES strain did not (Fig. 5). The MS spectrum then confirmed that the peak detected in pYES‐CbβAS transgenic strain was β‐amyrin (Fig. 6).

**Figure 7:**
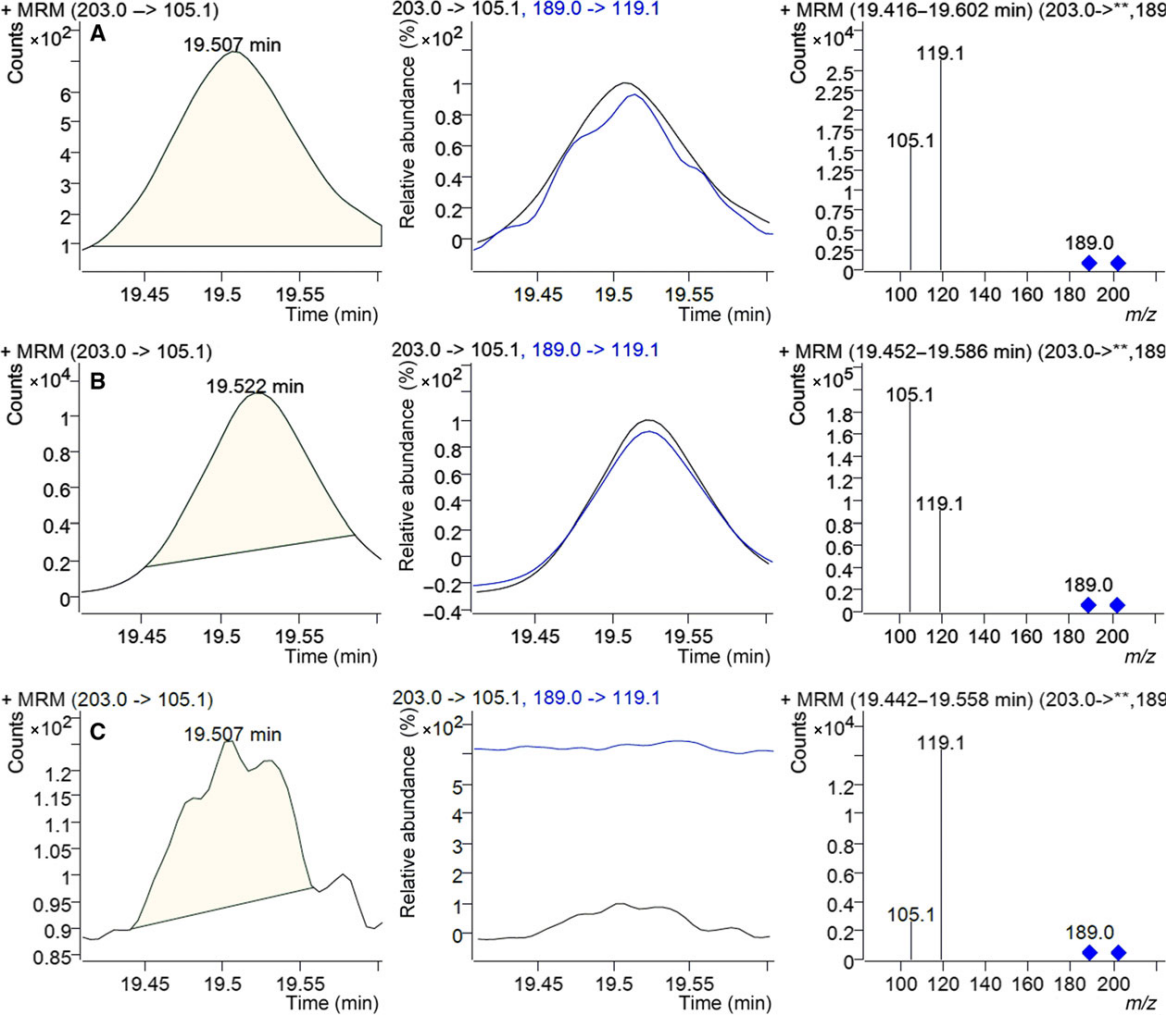
Multireaction monitor (MRM) analysis of 19.5‐min peak. (A) MRM analysis of β‐amyrin standard. (B) MRM analysis of pYES‐CbβAS yeast. (C) MRM analysis of pYES2/NT B control yeast.

GC‐MS/MS is an advanced detection system that provides high sensitivity for achieving very low detection thresholds. The precursor ion 203 *m/z* and daughter ion 105.1 *m/z* were used to detect β‐amyrin. Simultaneously precursor ion 189 *m/z* and daughter ion 119.1 *m/z* were used for quantification analysis (Fig. 7). The results showed that the pYES‐CbβAS yeast yielded 4.432 mg·L^−1^ β‐amyrin after induction by galactose for 60 h in 50 mL medium.

## Discussion

Currently, Chinese herbal medicine has become increasingly popular due to their abundant primary and secondary metabolites. These metabolites can be used to treat many diseases and have little side effects. However, the natural plants yield low contents of metabolites and require a long time to grow, which hampered the applications of the pharmacologically active compounds. Therefore, synthetic biology is an effective way to solve this contradiction 29. For example, the popular anticancer drug taxol 30, 31, 32 and the antimalarial drug artemisinin 33, 34, 35 are both successfully biosynthesized by microorganisms. The major pharmacological compound of *C. blinii* to be used in Chinese traditional medicine is conyzasaponins. However, there is a lack of information on the biosynthetic pathways of a majority of pharmacologically active compounds in *C. blinii*, especially conyzasaponins. In this study, we investigated this specific pathway by cloning and characterizing a *βAS* gene involved in it. To our knowledge, this is first study on conyzasaponins pathway.

Previous reports indicated that the DCTAE motif is highly conserved in eukaryotic OSCs. This motif is responsible for initiating the polycyclization reaction of squalene epoxide 36. The acidic carboxyl residue Asp in this motif releases protons to attack on the terminal epoxide ring of 1, which triggers a cascade of the ring‐forming reaction. The sequence analysis results of CbβAS suggest that it is an OSC. Besides, the MWCYCR is a characteristic motif of β‐amyrin synthase 37. In this motif, the Trp residue controls β‐amyrin formation by stabilization of oleanyl cation and the Tyr residue is involved in producing pentacyclic triterpenes. Therefore, the MWCYCR motif in CbβAS (Fig. 3) indicated that it is a special OSC, β‐amyrin synthase.

The preliminary functional verification of CbβAS is carried out by qRT‐PCR after the treatment of MeJA. Hayashi *et al*. 26 previously described that MeJA treatment can upregulate *βAS* mRNA levels and enhance the accumulation of soyasaponin (oleanane‐type triterpene saponin). Another report described by Liu *et al*. 38 also indicated that MeJA treatment upregulated the *Gentiana straminea βAS* expression levels and oleanolic acid accumulations. Conclusively, MeJA treatment can stimulate the accumulation of oleanane‐type saponins or sapogenins and the expression level of *βAS* gene. Therefore, if *CbβAS* is involved in the conyzasaponins pathway, its expression level will be upregulated by MeJA treatment. The qRT‐PCR results confirmed this conjecture that *CbβAS* is an enzyme involved in conyzasaponins formation.

We expressed *CbβAS* in *S. cerevisiae* to determine its function. GC‐MS/MS analysis showed that genetically engineered yeast with *CbβAS* produced 4.432 mg·L^−1^ β‐amyrin. Currently, the highest β‐amyrin titer achieved by microbial fermentation is 107.0 mg·L^−1^ 39. And the others indicated that by introducing βAS of *A. annua* 8 and *Pisum sativum* 40, the engineered *S. cerevisiae* produced 6 and 3.93 mg·L^−1^ β‐amyrin, respectively. The β‐amyrin yield of *CbβAS* transgenic yeast compared with earlier is not high. Modification of promoter and coexpression of genes involved in β‐amyrin pathway can be solutions to increase β‐amyrin contents.

In addition, further research on cytochrome P450 genes and glycosyltransferase genes involved in the conyzasaponins biosynthetic pathway is required to expand upon our results to utilize synthetic biology to produce conyzasaponins.

## Author contributions

HC and QW conceived and designed research. RS wrote the manuscript. SL provided *C. blinii* samples. ZZT and CLL contributed reagents or analytical tools. TRZ and TW performed the experiments. All authors read and approved the manuscript.

## References

[1] Su YF , Koike K , Guo D , Satou T , Liu JS , Zheng JH and Nikaido T (2001) New apiose‐containing triterpenoid saponins from *Conyza blinii* . Tetrahedron 57, 6721–6726.

[2] Su YF , Koike K , Nikaido T , Liu JS , Zheng JH and Guo D (2003) Conyzasaponins I‐Q, nine new triterpenoid saponins from *Conyza blinii* . J Nat Prod 66, 1593–1599. 14695803 10.1021/np030327o

[3] Su YF , Guo D , Guo HZ , Liu JS , Zheng JH , Koike K and Nikaido T (2001) Four new triterpenoid saponins from *Conyza blinii* . J Nat Prod 64, 32–36. 11170662 10.1021/np000310v

[4] Iturbe‐Ormaetxe I , Haralampidis K , Papadopoulou K and Osbourn AE (2003) Molecular cloning and characterization of triterpene synthases from *Medicago truncatula* and *Lotus japonicus* . Plant Mol Biol 51, 731–743. 12683345 10.1023/a:1022519709298

[5] Husselstein‐Muller T , Schaller H and Benveniste P (2001) Molecular cloning and expression in yeast of 2,3‐oxidosqualene‐triterpenoid cyclases from *Arabidopsis thaliana* . Plant Mol Biol 45, 75–92. 11247608 10.1023/a:1006476123930

[6] Wang Z , Guhling O , Yao R , Li F , Yeats TH , Rose JK and Jetter R (2011) Two oxidosqualene cyclases responsible for biosynthesis of tomato fruit cuticular triterpenoids. Plant Physiol 155, 540–552. 21059824 10.1104/pp.110.162883 PMC3075788

[7] Jin ML , Lee DY , Um Y , Lee JH , Park CG , Jetter R and Kim OT (2014) Isolation and characterization of an oxidosqualene cyclase gene encoding a beta‐amyrin synthase involved in *Polygala tenuifolia* Willd. saponin biosynthesis. Plant Cell Rep 33, 511–519. 24420413 10.1007/s00299-013-1554-7

[8] Kirby J , Romanini DW , Paradise EM and Keasling JD (2008) Engineering triterpene production in *Saccharomyces cerevisiae*‐beta‐amyrin synthase from *Artemisia annua* . FEBS J 275, 1852–1859. 18336574 10.1111/j.1742-4658.2008.06343.x

[9] Huang Z , Lin J , Cheng Z , Xu M , Guo M , Huang X , Yang Z and Zheng J (2015) Production of oleanane‐type sapogenin in transgenic rice via expression of beta‐amyrin synthase gene from *Panax japonicus* C. A. Mey. BMC Biotechnol 15, 45. 26033328 10.1186/s12896-015-0166-4 PMC4450844

[10] Paddon CJ , Westfall PJ , Pitera DJ , Benjamin K , Fisher K , McPhee D , Leavell MD , Tai A , Main A , Eng D *et al* (2013) High‐level semi‐synthetic production of the potent antimalarial artemisinin. Nature 496, 528–532. 23575629 10.1038/nature12051

[11] Engels B , Dahm P and Jennewein S (2008) Metabolic engineering of taxadiene biosynthesis in yeast as a first step towards Taxol (Paclitaxel) production. Metab Eng 10, 201–206. 18485776 10.1016/j.ymben.2008.03.001

[12] Han JY , Kim HJ , Kwon YS and Choi YE (2011) The Cyt P450 enzyme CYP716A47 catalyzes the formation of protopanaxadiol from dammarenediol‐II during ginsenoside biosynthesis in *Panax ginseng* . Plant Cell Physiol 52, 2062–2073. 22039120 10.1093/pcp/pcr150

[13] Sun R , Liu S , Tang ZZ , Jin HJ , Li CL and Chen H (2015) Study on transcriptome characteristic of genuine traditional Chinese medicine *Conyza blinii* H. Lév leaves of Sichuan. Mol Plant Breed 13, 2754–2760.

[14] Tamura K , Peterson D , Peterson N , Stecher G , Nei M and Kumar S (2011) MEGA5: molecular evolutionary genetics analysis using maximum likelihood, evolutionary distance, and maximum parsimony methods. Mol Biol Evol 28, 2731–2739. 21546353 10.1093/molbev/msr121 PMC3203626

[15] Sun R , Gao JL , Liu S , Tang ZZ , Li CL and Chen H (2013) Cloning and sequence analysis of glyceraldehyde‐3‐phosphate dehydrogenase gene from *Conyza blinii* . Chin Tradit Herbal Drugs 44, 2732–2735.

[16] Livak KJ and Schmittgen TD (2001) Analysis of relative gene expression data using real‐time quantitative PCR and the 2(‐Delta Delta C(T)) method. Methods 25, 402–408. 11846609 10.1006/meth.2001.1262

[17] Becker DM and Guarente L (1991) High‐efficiency transformation of yeast by electroporation. Methods Enzymol 194, 182–187. 2005786 10.1016/0076-6879(91)94015-5

[18] Seki H , Sawai S , Ohyama K , Mizutani M , Ohnishi T , Sudo H , Fukushima EO , Akashi T , Aoki T , Saito K *et al* (2011) Triterpene functional genomics in licorice for identification of CYP72A154 involved in the biosynthesis of glycyrrhizin. Plant Cell 23, 4112–4123. 22128119 10.1105/tpc.110.082685 PMC3246328

[19] Cammareri M , Consiglio MF , Pecchia P , Corea G , Lanzotti V , Ibeas JI , Tava A and Conicella C (2008) Molecular characterization of β‐amyrin synthase from *Aster sedifolius* L. and triterpenoid saponin analysis. Plant Sci 175, 255–261.

[20] Guhling O , Hobl B , Yeats T and Jetter R (2006) Cloning and characterization of a lupeol synthase involved in the synthesis of epicuticular wax crystals on stem and hypocotyl surfaces of *Ricinus communis* . Arch Biochem Biophys 448, 60–72. 16445885 10.1016/j.abb.2005.12.013

[21] Wang Z , Yeats T , Han H and Jetter R (2010) Cloning and characterization of oxidosqualene cyclases from *Kalanchoe daigremontiana*: enzymes catalyzing up to 10 rearrangement steps yielding friedelin and other triterpenoids. J Biol Chem 285, 29703–29712. 20610397 10.1074/jbc.M109.098871 PMC2943309

[22] Hu W , Liu N , Tian Y and Zhang L (2013) Molecular cloning, expression, purification, and functional characterization of dammarenediol synthase from *Panax ginseng* . Biomed Res Int 2013, 285740. 23484102 10.1155/2013/285740 PMC3591206

[23] Wang L , Zhao SJ , Cao HJ and Sun Y (2014) The isolation and characterization of dammarenediol synthase gene from *Panax quinquefolius* and its heterologous co‐expression with cytochrome P450 gene PqD12H in yeast. Funct Integr Genomics 14, 545–557. 24929308 10.1007/s10142-014-0384-1

[24] Abe I and Prestwich GD (1995) Identification of the active site of vertebrate oxidosqualene cyclase. Lipids 30, 231–234. 7791531 10.1007/BF02537826

[25] Poralla K , Hewelt A , Prestwich GD , Abe I , Reipen I and Sprenger G (1994) A specific amino acid repeat in squalene and oxidosqualene cyclases. Trends Biochem Sci 19, 157–158. 8016864 10.1016/0968-0004(94)90276-3

[26] Hayashi H , Huang P and Inoue K (2003) Up‐regulation of soyasaponin biosynthesis by methyl jasmonate in cultured cells of *Glycyrrhiza glabra* . Plant Cell Physiol 44, 404–411. 12721381 10.1093/pcp/pcg054

[27] Lee MH , Jeong JH , Seo JW , Shin CG , Kim YS , In JG , Yang DC , Yi JS and Choi YE (2004) Enhanced triterpene and phytosterol biosynthesis in *Panax ginseng* overexpressing squalene synthase gene. Plant Cell Physiol 45, 976–984. 15356323 10.1093/pcp/pch126

[28] Moses T , Pollier J , Almagro L , Buyst D , Van Montagu M , Pedreno MA , Martins JC , Thevelein JM and Goossens A (2014) Combinatorial biosynthesis of sapogenins and saponins in *Saccharomyces cerevisiae* using a C‐16 alpha hydroxylase from *Bupleurum falcatum* . Proc Natl Acad Sci USA 111, 1634–1639. 24434554 10.1073/pnas.1323369111 PMC3910630

[29] Chen SL , Zhun XX , Li CF , Wang Y , Yao H , Sun C and Song JY (2012) Genomics and synthetic biology of traditional Chinese medicine. Acta Pharm Sin 47, 1070–1078. 23162906

[30] Biggs BW , Lim CG , Sagliani K , Shankar S , Stephanopoulos G , De Mey M and Ajikumar PK (2016) Overcoming heterologous protein interdependency to optimize P450‐mediated Taxol precursor synthesis in *Escherichia coli* . Proc Natl Acad Sci USA 113, 3209–3214. 26951651 10.1073/pnas.1515826113 PMC4812725

[31] Guerra‐Bubb J , Croteau R and Williams RM (2012) The early stages of taxol biosynthesis: an interim report on the synthesis and identification of early pathway metabolites. Nat Prod Rep 29, 683–696. 22547034 10.1039/c2np20021j PMC3373433

[32] Jiang M , Stephanopoulos G and Pfeifer BA (2012) Downstream reactions and engineering in the microbially reconstituted pathway for Taxol. Appl Microbiol Biotechnol 94, 841–849. 22460591 10.1007/s00253-012-4016-1 PMC9896016

[33] Anthony JR , Anthony LC , Nowroozi F , Kwon G , Newman JD and Keasling JD (2009) Optimization of the mevalonate‐based isoprenoid biosynthetic pathway in *Escherichia coli* for production of the anti‐malarial drug precursor amorpha‐4,11‐diene. Metab Eng 11, 13–19. 18775787 10.1016/j.ymben.2008.07.007

[34] Dietrich JA , Yoshikuni Y , Fisher KJ , Woolard FX , Ockey D , McPhee DJ , Renninger NS , Chang MC , Baker D and Keasling JD (2009) A novel semi‐biosynthetic route for artemisinin production using engineered substrate‐promiscuous P450(BM3). ACS Chem Biol 4, 261–267. 19271725 10.1021/cb900006h

[35] Wang B , Kashkooli AB , Sallets A , Ting HM , deRuijter NC , Olofsson L , Brodelius P , Pottier M , Boutry M , Bouwmeester H *et al* (2016) Transient production of artemisinin in *Nicotiana benthamiana* is boosted by a specific lipid transfer protein from *A. annua* . Metab Eng 38, 159–169. 27421621 10.1016/j.ymben.2016.07.004

[36] Ito R , Masukawa Y and Hoshino T (2013) Purification, kinetics, inhibitors and CD for recombinant beta‐amyrin synthase from *Euphorbia tirucalli* L and functional analysis of the DCTA motif, which is highly conserved among oxidosqualene cyclases. FEBS J 280, 1267–1280. 23294602 10.1111/febs.12119

[37] Kushiro T , Shibuya M , Masuda K and Ebizuka Y (2000) Mutational studies on triterpene synthases: engineering lupeol synthase into β‐amyrin synthase. J Am Chem Soc 122, 6816–6824.

[38] Liu YL , Cai YF , Zhao ZJ , Wang JF , Li J , Xin W , Xia GM and Xiang FN (2009) Cloning and functional analysis of a beta‐amyrin synthase gene associated with oleanolic acid biosynthesis in *Gentiana straminea* MAXIM. Biol Pharm Bull 32, 818–824. 19420748 10.1248/bpb.32.818

[39] Dai ZB , Wang BB , Liu Y , Shi MY , Wang D , Zhang XN , Liu T , Huang LQ and Zhang XL (2014) Producing aglycons of ginsenosides in bakers’ yeast. Sci Rep 4, 3698. 24424342 10.1038/srep03698 PMC3892717

[40] Madsen KM , Udatha GD , Semba S , Otero JM , Koetter P , Nielsen J , Ebizuka Y , Kushiro T and Panagiotou G (2011) Linking genotype and phenotype of *Saccharomyces cerevisiae* strains reveals metabolic engineering targets and leads to triterpene hyper‐producers. PLoS One 6, e14763. 21445244 10.1371/journal.pone.0014763 PMC3060802

